# Identification and characterization of a small molecule BFstatin inhibiting BrpR, the transcriptional regulator for biofilm formation of *Vibrio vulnificus*

**DOI:** 10.3389/fmicb.2024.1468567

**Published:** 2024-09-09

**Authors:** Hojun Lee, Seung-Ho Hwang, Hyunwoo Shin, Nam-Chul Ha, Qiyao Wang, Sang Ho Choi

**Affiliations:** ^1^Department of Agricultural Biotechnology, National Research Laboratory of Molecular Microbiology and Toxicology, Seoul National University, Seoul, Republic of Korea; ^2^Department of Agricultural Biotechnology, Seoul National University, Seoul, Republic of Korea; ^3^Center for Food and Bioconvergence, Seoul National University, Seoul, Republic of Korea; ^4^State Key Laboratory of Bioreactor Engineering, East China University of Science and Technology, Shanghai, China; ^5^Research Institute of Agriculture and Life Sciences, Seoul National University, Seoul, Republic of Korea

**Keywords:** biofilm, inhibitor, transcriptional regulator, pathogen, *Vibrio vulnificus*

## Abstract

Many pathogenic bacteria form biofilms that are resistant to not only host immune defenses but also antibiotics, posing a need for the development of strategies to control biofilms. In this study, to prevent biofilm formation of the fulminating foodborne pathogen *Vibrio vulnificus*, chemical libraries were extensively screened to identify a small molecule inhibiting the activity of BrpR, a transcriptional regulator for biofilm genes. Accordingly, the BrpR inhibitor BFstatin [N1-(2-chloro-5-fluorophenyl)-N3-propylmalonamide], with a half-maximal effective concentration of 8.01 μM, was identified. BFstatin did not interfere with bacterial growth or exhibit cytotoxicity to the human epithelial cell line. BFstatin directly bound to BrpR and interrupted its binding to the target promoter DNAs of the downstream genes. Molecular dynamics simulation of the interaction between BFstatin and BrpR proposed that BFstatin modifies the structure of BrpR, especially the DNA-binding domain. Transcriptomic analyses revealed that BFstatin reduces the expression of the BrpR regulon including the *cabABC* operon and *brp* locus which contribute to the production of biofilm matrix of *V. vulnificus*. Accordingly, BFstatin diminished the biofilm levels of *V. vulnificus* by inhibiting the matrix development in a concentration-dependent manner. Altogether, BFstatin could be an anti-biofilm agent targeting BrpR, thereby rendering *V. vulnificus* more susceptible to host immune defenses and antibiotics.

## 1 Introduction

Bacteria often form biofilms which are surface-attached microbial communities (O’toole et al., 2000). Biofilm formation includes sequential developmental stages composed of attachment to the surface, formation of microcolony, maturation into three-dimensional structures, and dispersal of bacterial cells from mature biofilms (Watnick and Kolter, 2000). Mature biofilms are highly differentiated communities of bacteria covered by an extracellular polymeric matrix consisting of exopolysaccharides (EPSs), proteins, nucleic acids, and lipids (Flemming and Wingender, 2010; Mai-Prochnow et al., 2021). The biofilm matrix provides pathogenic bacteria with protection from host immune defenses and antibiotics, resulting in enhanced survival and virulence in the course of infection (Stewart and Costerton, 2001; Hall-Stoodley et al., 2004; Hall-Stoodley and Stoodley, 2005; Flemming et al., 2016). Strategies currently used to control biofilms of pathogenic bacteria are mostly based on antibiotic treatment (Koo et al., 2017). However, completely eradicating the biofilm using the control strategies is difficult because bacteria within the biofilms are highly resistant to antibiotics (Costerton et al., 1999; Hoiby et al., 2010; Koo et al., 2017). Therefore, effective methods to prevent biofilm formation are urgently required to treat bacterial infection. The methods should not inhibit bacterial growth, because the inhibition of viability can lead to the dominance of resistant strains (Srinivasan et al., 2021).

*Vibrio vulnificus*, a fulminating foodborne pathogen, forms biofilm to survive and persist in seafood such as oysters, the major infection route of the bacterium (Froelich and Oliver, 2013; Kim et al., 2013; Pu et al., 2018; Choi and Choi, 2022). Several studies have been carried out to understand the molecular mechanisms of *V. vulnificus* to form biofilm (Guo and Rowe-Magnus, 2010; Park et al., 2015a, 2015b; Chodur and Rowe-Magnus, 2018; Hwang et al., 2020, 2021; Lee et al., 2023). A transcription factor BrpR regulates, directly and indirectly, the expression of genes required for biofilm formation. BrpR directly activates the expression of *brpLG* and *brpT*, according to the intracellular levels of bis-(3′-5′)-cyclic dimeric guanosine monophosphate (c-di-GMP), a universal bacterial second messenger (Park et al., 2015b; Hwang et al., 2021). BrpL and BrpG are involved in the production of EPS, which is a key component of the biofilm matrix (Hwang et al., 2021). BrpT, another transcriptional regulator, activates the expression of the *cabABC* operon, *cabH*, the *brp* locus (*brpABCDFHIJK*), *brpN*, and *brpS* (Chodur and Rowe-Magnus, 2018; Hwang et al., 2020; Lee et al., 2023). CabA, secreted to the cell exterior through the CabBC secretion system, forms a structure of the biofilm matrix (Park et al., 2015a). CabH, also predicted to be a matrix component, contributes to the surface attachment of *V. vulnificus* (Lee et al., 2023). Products of the *brp* locus and *brpN* participate in EPS production in concert with BrpL and BrpG (Guo and Rowe-Magnus, 2010; Hwang et al., 2021; Lee et al., 2023). BrpS is also a transcriptional regulator that activates *cabABC* and represses *brpT* expression to constitute a negative feedback loop tuning the *brpT* expression level precisely (Hwang et al., 2020). In summary, BrpR is a master regulator that directly controls the expression of *brpLG* and indirectly the *cabABC* operon, *cabH*, the *brp* locus, and *brpN* involved in the biofilm matrix development of *V. vulnificus*. Therefore, inhibiting the activity of BrpR will eventually lead to the prevention of biofilm formation of *V. vulnificus*.

In this study, a high-throughput screening of 6,750 compounds was performed to identify a small-molecule inhibitor of *V. vulnificus* BrpR. As a result, BFstatin, which significantly decreased the activity of BrpR in a dose-dependent manner, was identified. BFstatin did not affect the growth of *V. vulnificus* or show cytotoxicity to the human epithelial cell line. BFstatin directly interacted with BrpR, inhibiting its binding to the target promoter DNAs. Moreover, molecular dynamics (MD) simulation showed that the interaction of BFstatin with BrpR alters the structure of BrpR to lessen its DNA-binding activity. RNA sequencing analyses revealed that BFstatin reduced the expression of the BrpR regulon involved in the biofilm matrix development. In conclusion, this study identified a small molecule BFstatin and characterized its molecular mechanism to control the activity of BrpR which is essential for the biofilm formation of *V. vulnificus*.

## 2 Materials and methods

### 2.1 Strains, plasmids, and culture conditions

The strains and plasmids used in this study are listed in Supplementary Table S1. Unless otherwise noted, *Escherichia coli* and *V. vulnificus* strains were grown aerobically in Luria-Bertani (LB) medium and LB supplemented with 2% (w/v) NaCl (LBS) at 37°C and 30°C, respectively. For biofilm formation, the *Vibrio fischeri* minimal medium containing glycerol (50 mM Tris-HCl, pH 7.2, 50 mM MgSO_4_, 300 mM NaCl, 10 mM KCl, 0.33 mM K_2_HPO_4_, 18.5 mM NH_4_Cl, 10 mM CaCl_2_, and 32.6 mM glycerol) (VFMG) was used (Hwang et al., 2021). JN111, the *V. vulnificus* CMCP6 strain, which carries *dcpA* encoding a diguanylate cyclase (Nakhamchik et al., 2008) on the chromosome under the control of the arabinose-inducible promoter P_BAD_ (Guzman et al., 1995), was used as a model strain in this study (Supplementary Table S1). The intracellular c-di-GMP levels of JN111 and its mutant strain were manipulated by adding different concentrations of arabinose to the growth media. HeLa cells originated from the American Type Culture Collection were maintained at 37°C with 5% CO_2_ in Dulbecco’s modified Eagle’s medium containing 10% fetal bovine serum, 50 μg/mL penicillin, and 50 μg/mL streptomycin.

### 2.2 High-throughput screening

For high-throughput screening, a random chemical library consisting of small molecules selected based on structural diversity and drug-likeness and then dissolved in 100% DMSO at 1 mM was obtained from the Korea Chemical Bank^1^ and used. The *brpR* ORF, amplified by PCR with appropriate primer pairs (Supplementary Table S2), was subcloned into pJK1113 (Lim et al., 2014) under P_BAD_ to yield pJN1601 (Supplementary Table S1). The promoter DNA of VV1_2288, P_VV1_2288_, was amplified by PCR with appropriate primer pairs (Supplementary Table S2) and then fused to the promoterless *lux* operon of pBBR-lux (Lenz et al., 2004) to create pSH2103, a BrpR-repressible reporter plasmid (Supplementary Table S2). *E. coli* DH5α was cotransformed with pJN1601 and pSH2103 to create a reporter strain. The *E. coli* reporter strain was grown to *A*_600_ of 0.5 in LB containing 0.0002% (w/v) L-(+) arabinose, 20 μg/mL chloramphenicol, and 100 μg/mL ampicillin. An aliquot (98 μL) of the culture was transferred to each well of a 96-well black microtiter plate (Nunc, Roskilde, Denmark) containing 2 μL of the small molecules to achieve 20 μM of each molecule or 2% DMSO (control) and incubated at 37°C with shaking. After 4 h incubation, luminescence and growth (absorbance at 600 nm, *A*_600_) of the reporter strain in each well were measured using a Spark microplate reader (Tecan, Männedorf, Switzerland), and relative luminescence units (RLUs) were calculated by dividing luminescence with *A*_600_. Hit molecules inhibiting more than 20% of the BrpR activity were selected for further verification.

### 2.3 Verification of hit molecules and determination of the half-maximal effective concentration (EC_50_) of BFstatin

The *brpT* promoter, P*_brpT_*, was amplified by PCR with appropriate primer pairs (Supplementary Table S2) and then fused to the promoterless *lux* operon of pBBR-lux to create pJN1606, a BrpR-inducible reporter plasmid (Supplementary Table S1). *V. vulnificus* JN111 and the isogenic *brpR* mutant conjugally received either pSH2103 or pJN1606 to create reporter strains. The *V. vulnificus* reporter strains were grown to *A*_600_ of 0.5 in LBS containing 3 μg/mL chloramphenicol. Then the hit molecules (20 μM) were treated to the culture and RLUs were calculated after 4 h incubation as described above.

To determine the EC_50_ of BFstatin, the *V. vulnificus* reporter strain containing pJN1606 was grown to *A*_600_ of 0.5 in LBS containing 3 μg/mL chloramphenicol. Then various concentrations (to make final concentrations of 10^−10^ to 10^−4^ M) of BFstatin were treated to the culture and RLUs were calculated after 4 h incubation as described above. The relative BrpR activities were expressed using the RLU observed in the absence of BFstatin as 100% and the RLU of Δ*brpR* as 0%. The EC_50_ was calculated by plotting the relative BrpR activities versus the BFstatin concentration using GraphPad Prism 9.0 (GraphPad Software, San Diego, CA, United States).

### 2.4 Lactate dehydrogenase release assay

To examine the cytotoxicity of BFstatin, the activity of cytoplasmic lactate dehydrogenase (LDH) which is released from damaged cells was measured as an indicator of cell damage using the LDH Cytotoxicity Detection Kit (Takara, Tokyo, Japan). The monolayers of HeLa cells grown in a 96-well tissue culture plate (Nunc) were treated with either 20 or 100 μM BFstatin or 1% DMSO (control). After 3 h incubation at 37°C, the LDH activities in the supernatant were evaluated by measurement of absorbance at 490 nm as described previously (Kumar et al., 2018).

### 2.5 Protein purification, microscale thermophoresis, and electrophoretic mobility shift assay

To overexpress BrpR, pSH1820 carrying the *brpR* gene on pET-28a(+) (Novagen, Madison, WI, United States) was used as described previously (Hwang et al., 2021). The His6-tagged BrpR was expressed in *E. coli* BL21 (DE3) and purified by affinity chromatography using Ni-NTA agarose (Qiagen, Valencia, CA, United States).

For microscale thermophoresis (MST), BrpR was labeled using the Monolith His-tag Labeling Kit RED-tris-NTA 2nd Generation (NanoTemper Technologies, Munich, Germany). Labeled BrpR (final concentration of 50 nM) was mixed with various concentrations of BFstatin (to make final concentrations of 2^−16^ to 2^−1^ mM) in 1 × BrpR-binding buffer for MST (40 mM Tris-Cl, pH 7.9, 100 mM KCl, 10 mM MgCl_2_, 1 mM DTT, and 0.05% Tween-20). MST was performed in a Monolith NT.115 Pico (NanoTemper Technologies) using the Monolith NT.115 Premium Capillaries (NanoTemper Technologies).

For electrophoretic mobility shift assay (EMSA), promoter DNAs of VV1_2288 (258-bp P_VV1_2288_), *brpL* (301-bp P*_brpL_*), and *brpR* (294-bp P*_brpR_*) were amplified by PCR using appropriate primer pairs (Supplementary Table S2). The resulting 6-FAM-labeled DNAs (5 nM) were incubated with different amounts of purified BrpR for 2 h at 30°C in a 20 μL reaction mixture containing 1 × BrpR-binding buffer for EMSA (40 mM Tris-Cl, pH 7.9, 100 mM KCl, 10 mM MgCl_2_, 1 mM DTT, 0.1 mM EDTA, 50 μM c-di-GMP, and 0.1 μg/μL bovine serum albumin) and 0.1 μg of poly (dI-dC) as a non-specific competitor. In the experiment with BFstatin, DMSO was added to the reaction mixture to a final concentration of 20%. For the competition analyses, various concentrations of unlabeled DNA fragments were added as a self-competitor to the reaction mixture before incubation. Electrophoretic analysis for the DNA-protein complexes was performed as described previously (Lee et al., 2020; Ko et al., 2023).

### 2.6 Protein structure prediction, molecular docking, and MD simulation

The structural prediction of the full-length BrpR dimer was obtained using the AlphaFold2 algorithm (Jumper et al., 2021). Visualization of the structures of BrpR dimer and BFstatin was conducted using PyMOL (DeLano, 2002). Molecular docking of BrpR dimer and BFstatin was performed using AutoDock Vina in PyRx virtual screening software (Dallakyan and Olson, 2015). The docked BrpR-BFstatin complex was analyzed using MD simulation with Gromacs software (Hess et al., 2008; Pronk et al., 2013). The topology file of the BrpR-BFstatin complex used in the MD simulation was prepared using the CHARMM36m force field in CHARMM-GUI (Jo et al., 2008; Huang et al., 2017). Before the MD simulation, the BrpR-BFstatin complex was solvated in TIP3P water and neutralized by adding 150 mM NaCl. The BrpR-BFstatin complex was energy-minimized using the steepest descent algorithm and then equilibrated with 125 ps NVT and NPT simulation to attain a temperature of 303 K and pressure of 1 bar, respectively. The resulting BrpR-BFstatin complex was regarded as the refined structure at 0 ns. Then the MD simulation was carried out for 100 ns with the time step of 0.002 ps.

### 2.7 RNA sequencing and analysis

To extract RNA, each well of the 24-well microtiter plates (SPL, Seoul, Republic of Korea) was inoculated using 1 mL of culture diluted to an *A*_600_ of 0.8 in VFMG supplemented with 0.01% (w/v) arabinose and either 20 μM BFstatin or 2% DMSO (control). After static incubation for 2 h at 30°C, total RNA was isolated from the bacterial cells using an RNeasy mini kit (Qiagen). mRNA sequencing libraries were prepared using the Truseq stranded mRNA library prep kit (Illumina, San Diego, CA, United States). mRNA was purified and fragmented from total RNA (1 μg) using poly-T oligo-attached magnetic beads. The fragmented RNAs were primed with random hexamers and reverse transcribed into first-strand cDNA using reverse transcriptase and dUTP in place of dTTP. The first-strand cDNA fragments were then added with single “A” bases and ligated with the adapter. The resulting cDNA fragments were purified and amplified by PCR to create the final cDNA library. The cDNA library was clustered in a flow cell on the cBot automated cluster generation system (Illumina). Then the flow cell was loaded on NovaSeq 6000 system (Illumina), and sequencing was performed with 2 × 100 bp read length. The raw sequencing reads were mapped onto the *V. vulnificus* CMCP6 reference genome (GenBank accession numbers NC004459.3 and NC004460.2) using Kallisto (Bray et al., 2016). The expression level of each gene was defined using transcripts per million (TPM) and average log counts per million (logCPM). Quantile-normalized TPM values were then statistically analyzed by student’s *t*-tests to identify the genes that were differentially expressed (|fold change| >2, adjusted *p*-value <0.05, and logCPM ≥1). All raw transcriptome data have been deposited in the NCBI BioProject database^2^ under accession number PRJNA1138499.

### 2.8 EPS analysis

EPS was prepared following the procedures described previously (Kim et al., 2007). Briefly, each culture grown on an LBS agar containing 0.02% (w/v) arabinose and either 20 or 100 μM BFstatin or 0.1% DMSO (control) was scraped and suspended in phosphate-buffered saline (PBS) and diluted to an *A*_600_ of 1.0. The suspensions were vigorously shaken to elute the EPS from the cells. The cells and debris were removed by centrifugation, and the supernatant was treated with RNase A (50 μL/mL), DNase I (50 μg/mL with 10 mM MgCl_2_), and proteinase K (200 μg/mL). Subsequently, the remaining EPS fraction was extracted with phenol-chloroform, precipitated with 2.5 × volumes of ethanol, and resuspended in distilled water. The EPS resuspensions were resolved on a 4% polyacrylamide gel by SDS-PAGE and stained with Stains-All (Sigma-Aldrich, St. Louis, MO, United States). The gel was subsequently destained as described previously (Kelley and Parker, 1981) and photographed by a mobile camera. The intensity of stained EPS in each lane with BFstatin was determined using ImageJ software (NIH, Bethesda, MD, United States), and then compared to that with 0.1% DMSO (control).

### 2.9 Colony morphology assay

For the analysis of the colony morphology, 2 μL of cultures grown to an *A*_600_ of 0.8 were spotted onto VFMG agar supplemented with 0.02% (w/v) arabinose and either 20 or 100 μM BFstatin or 0.1% DMSO (control). The resulting colonies grown at 30°C for 24 h were visualized using a Stemi 305 stereomicroscope (Zeiss, Oberkochen, Germany) equipped with an Axiocam 105 color camera (Zeiss).

### 2.10 Quantification and visualization of the biofilms

To quantify the biofilms of *V. vulnificus*, each well of the 96-well polystyrene microtiter plates (Nunc) was inoculated using 200 μL of culture diluted to an *A*_600_ of 0.05 in VFMG supplemented with 0.01% (w/v) arabinose and either 20 or 100 μM BFstatin or 2% DMSO (control). After static incubation for 6, 12, 18, 24, and 30 h at 30°C, supernatants were removed from the wells, and the remaining biofilms were stained with 1% (w/v) crystal violet solution for 15 min. Then the biofilms were quantified by elution of the crystal violet with ethanol and measurement of absorbance at 570 nm (*A*_570_) as described previously (Ko and Choi, 2021). To visualize the biofilms, biofilms of *V. vulnificus* were formed and stained as explained above but in a larger scale (1 mL) using glass test tubes. To remove loosely attached cells, the biofilms were washed with a vibration of 1,200 rpm for 20 s in 1 mL of PBS. The remaining biofilms were photographed by a mobile camera after being stained and washed, respectively.

### 2.11 Data analysis

Average and standard deviation (SD) values were calculated from at least three independent experiments. The experimental data were analyzed by Student’s *t* tests using GraphPad Prism 9.0. The significance of the differences between experimental groups was accepted at a *p*-value of <0.05.

## 3 Results

### 3.1 Small molecules interfere with BrpR

**Figure 1:**
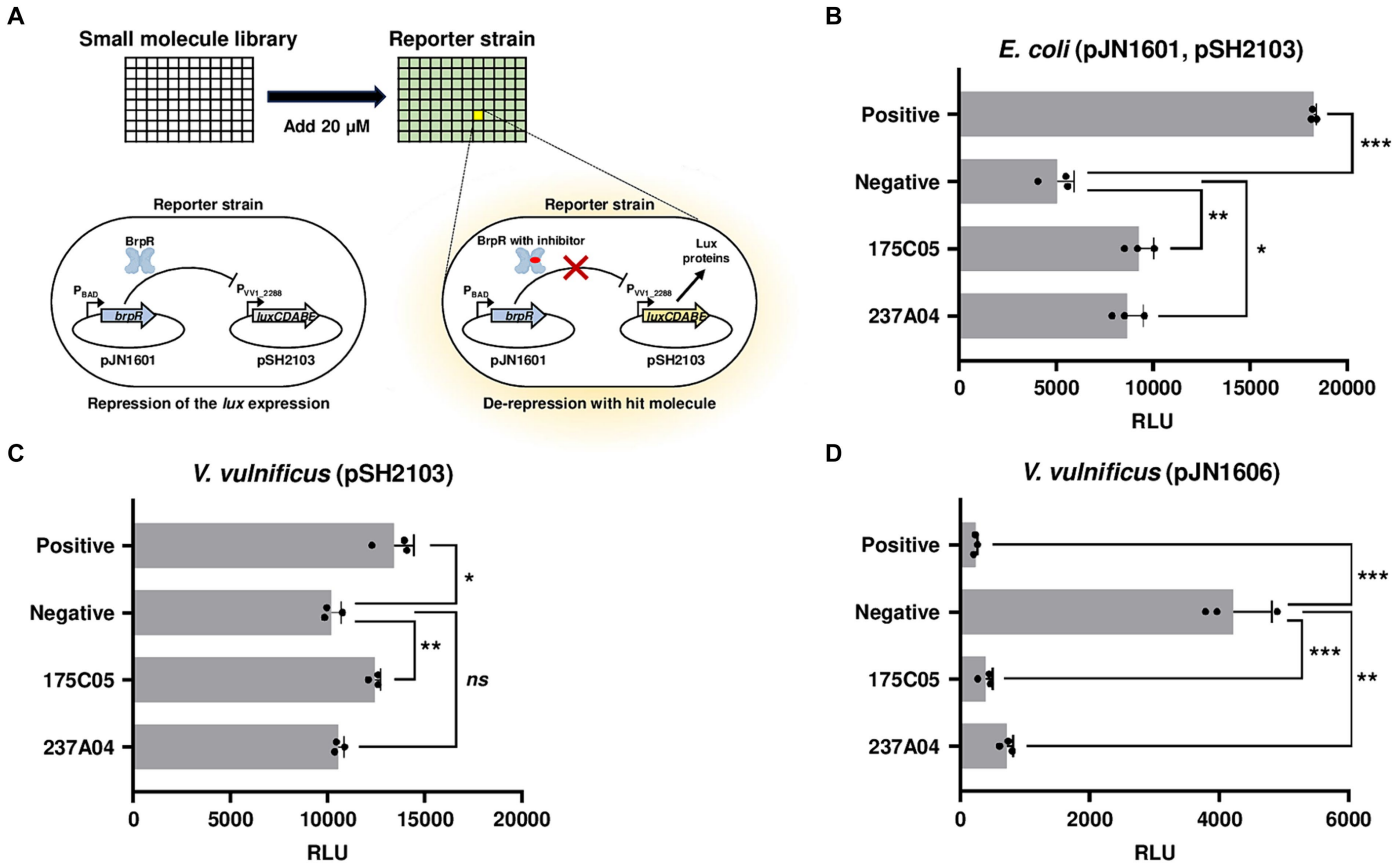
High-throughput screening for BrpR inhibitors. **(A)** A schematic demonstration of high-throughput screening of small molecules. An *E. coli* reporter strain contains pJN1601 expressing BrpR under arabinose-inducible promoter P_BAD_ and pSH2103 carrying the *luxCDABE* genes under BrpR-repressible promoter P_VV1_2288_. RLUs of the reporter strain were observed after the 20 μM addition of small molecules. **(B–D)** Each bar represents the RLU of *E. coli* containing pJN1601 and pSH2103 **(B)**, *V. vulnificus* containing pSH2103 **(C)**, and *V. vulnificus* containing pJN1606 carrying the *luxCDABE* genes under BrpR-inducible promoter P*_brpT_* **(D)** in the presence of hit molecules as indicated. Error bars represent the SD from biological triplicates. Statistical significance was determined by the student’s *t*-test (^*^p < 0.05, ^**^p < 0.005, and ^***^p < 0.0005; ns, not significant). Positive, RLUs from *E. coli* without arabinose **(B)** or *V. vulnificus* JN111 *brpR* mutant **(C,D)**; negative, RLUs from *E. coli* with arabinose **(B)** or *V. vulnificus* JN111 **(C,D)**; RLU, relative luminescence unit.

To identify a specific inhibitor for biofilm formation of *V. vulnificus*, transcriptional regulator BrpR was selected as an inhibitory target. An *E. coli* reporter strain containing pJN1601 (*brpR* is expressed by P_BAD_, an arabinose-inducible promoter) and a reporter plasmid pSH2103 (carrying a promoterless *lux* operon fused to a promoter P_VV1_2288_) was constructed (Figure 1A). The possible factors other than BrpR affecting the activity of P_VV1_2288_ in *V. vulnificus* could be removed by using the *E. coli* reporter strain. Because the P_VV1_2288_ is directly repressed by BrpR (Hwang et al., 2021) (Supplementary Figure S1A), the reporter strain remains non-luminescent in arabinose-containing media unless a potential hit molecule inhibits either the expression or activity of BrpR (Figure 1A). By using the BrpR-repressible reporter system instead of the BrpR-inducible system, the false identification of the molecules obstructing luminescence itself as hits could be excluded. Screening a random chemical library containing 6,696 molecules identified two compounds, 175C05 and 237A04, as hit molecules inhibiting either the expression or activity of BrpR (Figure 1B; Supplementary material S1). Based on the structures of the two hit molecules, 54 compounds with similar structures were additionally examined using the reporter system. However, their effect inhibiting the expression or activity of BrpR was lower than that of 175C05 and 237A04 (Supplementary material S2).

To distinguish whether 175C05 and 237A04 inhibit either the expression or activity of BrpR, a *V. vulnificus* reporter strain, in which *brpR* is chromosomally expressed by its own promoter, was constructed. 175C05 increased the luminescence of *V. vulnificus* containing the reporter plasmid pSH2103, while 237A04 had no significant effect (Figure 1C). Since 175C05 influenced luminescence in both *E. coli* and *V. vulnificus* reporter strains, in which *brpR* was expressed under different promoters (P_BAD_ and its own promoter), it is reasonable to assume that 175C05 inhibited the activity rather than the expression of BrpR. To further confirm the BrpR-inhibiting activity of 175C05, the *V. vulnificus* reporter strain containing a reporter plasmid pJN1606 was constructed. In contrast to pSH2103, pJN1606 carries the promoterless *lux* operon fused to the *brpT* promoter, P*_brpT_*, which is directly induced by BrpR (Hwang et al., 2021). 175C05 drastically reduced the luminescence of *V. vulnificus* containing pJN1606 (Figure 1D), validating that 175C05 inhibits the activity of BrpR. 237A04 also diminished the luminescence of *V. vulnificus* containing pJN1606, but the effect was weaker than that of 175C05 (Figure 1D). Together, 175C05 was finally identified as a putative small molecule inhibiting the activity of BrpR.

### 3.2 The BrpR inhibitor BFstatin impedes the activity of BrpR

**Figure 2:**
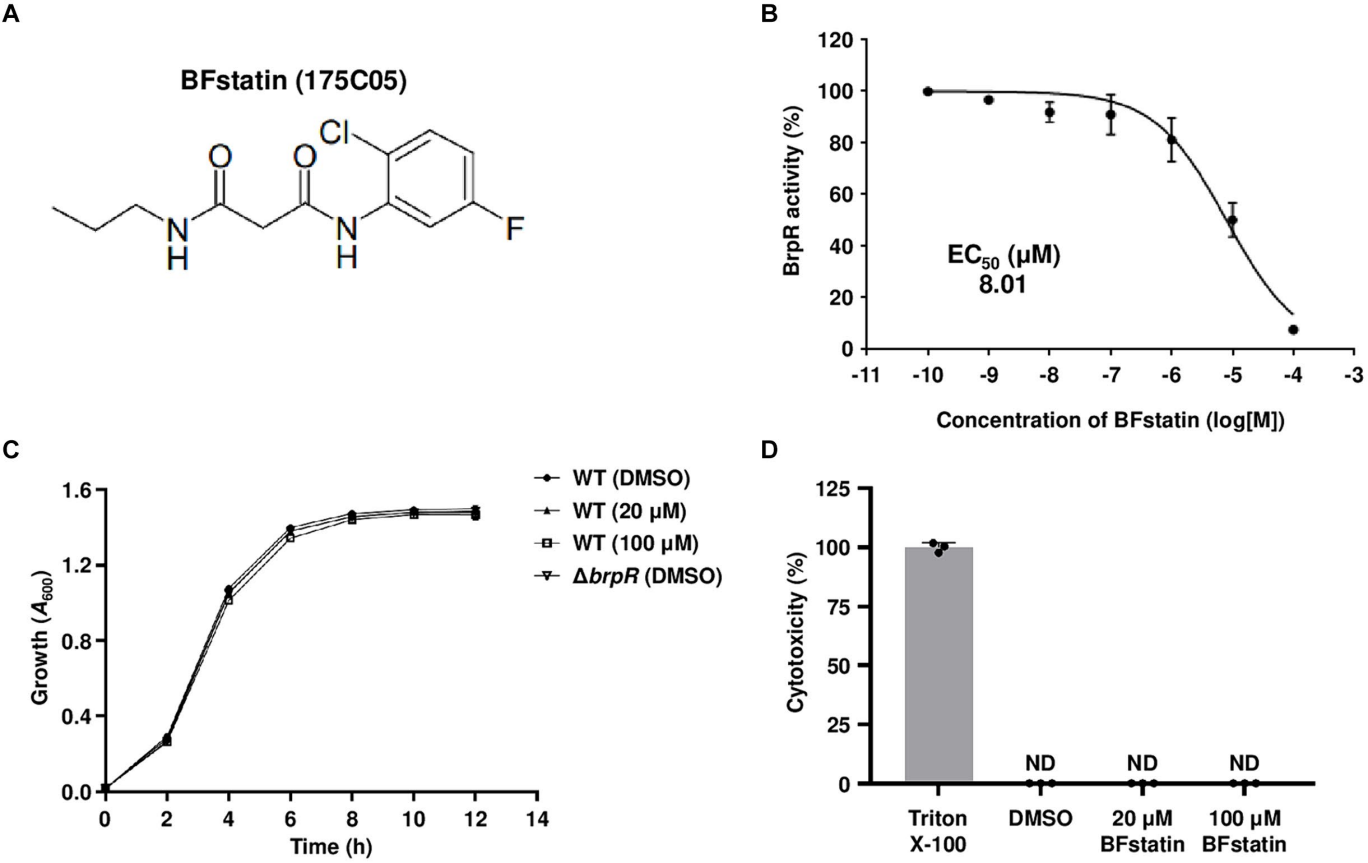
Effects of BFstatin on the BrpR activity, bacterial growth, and human cell viability. **(A)** The chemical structure of BFstatin, N1-(2-chloro-5-fluorophenyl)-N3-propylmalonamide. **(B)** The EC_50_ of BFstatin inhibiting the relative BrpR activity (%) was calculated as described in the *Materials and methods* section. **(C)** Growth of the *V. vulnificus* strains along with either 20 or 100 μM BFstatin or 2% DMSO (control) was monitored at 2 h intervals using a microplate reader and expressed as *A*_600_. WT, *V. vulnificus* CMCP6; Δ*brpR*, *V. vulnificus* CMCP6 *brpR* mutant. **(D)** The relative cytotoxicity (%) of BFstatin was determined using LDH activities released from HeLa cells incubated at 37°C for 3 h with either 20 or 100 μM BFstatin or 2% DMSO (control). The cytotoxicity was expressed using the LDH activity from the cells completely lysed by 4% Triton X-100 as 100%. ND, not detected. Error bars represent the SD from biological triplicates **(B,C)** or the representative of three independent experiments **(D)**.

The chemical structure of 175C05, N1-(2-chloro-5-fluorophenyl)-N3-propylmalonamide, is shown in Figure 2A and its molecular weight is 272.71 g/mol. To determine the EC_50_ of 175C05, BrpR activities were assessed using *V. vulnificus* containing pJN1606 in the presence of 0.01% arabinose and various concentrations of 175C05. Consequently, the EC_50_ of 175C05 was determined as 8.01 μM (Figure 2B), demonstrating that the chemical effectively inhibits the activity of BrpR at low doses in the micromolar range. Remarkably, 175C05 did not impede the growth of *V. vulnificus* up to 100 μM (Figure 2C), implying that the molecule minimizes the dominance of resistant strains. In addition, 175C05 was not cytotoxic to the human epithelial HeLa cells up to 100 μM (Figure 2D), broadening its application in the seafood industry. Altogether, the results indicated that 175C05 is a small-molecule inhibitor of BrpR activity that could be developed into an anti-biofilm agent against *V. vulnificus*. Subsequently, 175C05 was renamed as “BFstatin.”

### 3.3 BFstatin interacts with BrpR to inhibit its binding to target promoter DNA

**Figure 3:**
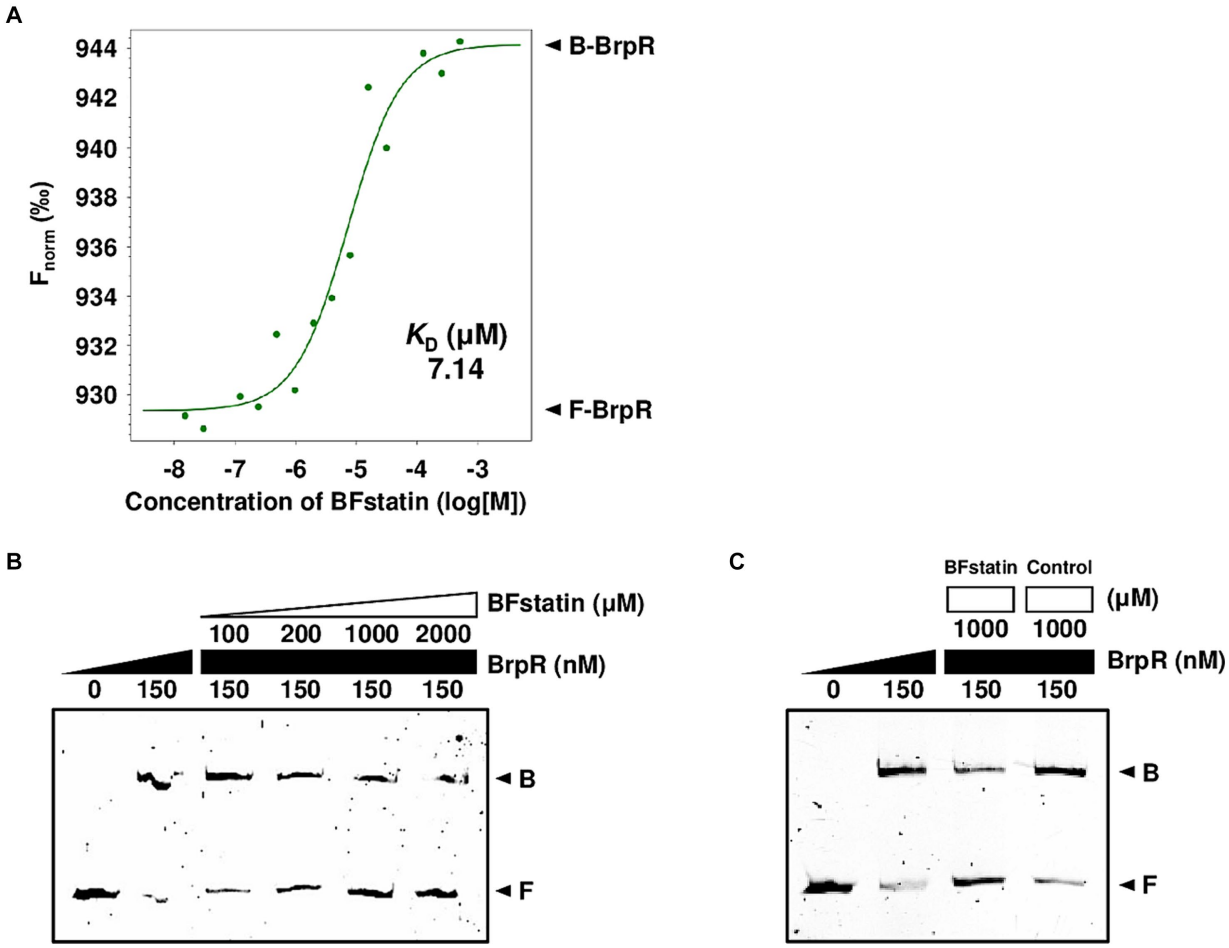
Molecular interactions between BrpR, BFstatin, and promoter DNA. **(A)** The *K*_D_ between BrpR and BFstatin was determined by MST as described in the *Materials and methods* section. F_norm_, normalized fluorescence; B-BrpR, BFstatin-bound BrpR; F-BrpR, free BrpR. **(B,C)** The 6-FAM labeled *brpL* promoter DNA probe (5 nM) was incubated with increasing amounts of BrpR (from 0 to 150 nM) and BFstatin (**B**, from 0 to 2,000 μM; **C**, from 0 to 1,000 μM) as indicated. A random small molecule (control) that showed no BrpR-inhibiting activity was added instead of BFstatin **(C)**. Each gel representing the mean result from at least three independent experiments was photographed using the ChemiDoc Touch Imaging System. B, BrpR-bound DNA; F, free DNA.

Since BFstatin reduced the activity of BrpR, BFstatin might interact with BrpR directly. To examine whether BFstatin directly interacts with BrpR, MST was performed. Normalized fluorescence (F_norm_) of fluorescence-labeled BrpR was gradually increased by BFstatin in a dose-dependent manner and a binding curve was obtained with a dissociation constant (*K*_D_) of 7.14 μM, verifying that BFstatin interacts directly with BrpR (Figure 3A).

As a transcriptional regulator, BrpR functions by binding directly to its target promoter DNA (Hwang et al., 2021). To examine whether BFstatin inhibits the DNA-binding activity of BrpR, EMSAs were performed with the labeled *brpL* promoter (P*_brpL_*) DNA probe. The addition of BrpR to the labeled DNA probe resulted in retarded bands (Figure 3B), confirming the previous observation that BrpR binds directly to P*_brpL_* DNA^17^. The addition of increasing amounts of BFstatin to the BrpR-P*_brpL_* DNA mixture led to a concentration-dependent reduction of the retarded DNA bands (Figure 3B), indicating that BFstatin inhibits the binding of BrpR to its target P*_brpL_* DNA. Additionally, the reduction of the retarded band obtained by the addition of 1,000 μM BFstatin was not achieved by the addition of the same amount of a random chemical (Figure 3C), indicating that the BFstatin inhibition of the BrpR binding to DNA is specific. Together, the results demonstrated that BFstatin directly interacts with BrpR and specifically inhibits the DNA-binding activity of BrpR to its target DNA.

### 3.4 BFstatin modifies the structure of BrpR

**Figure 4:**
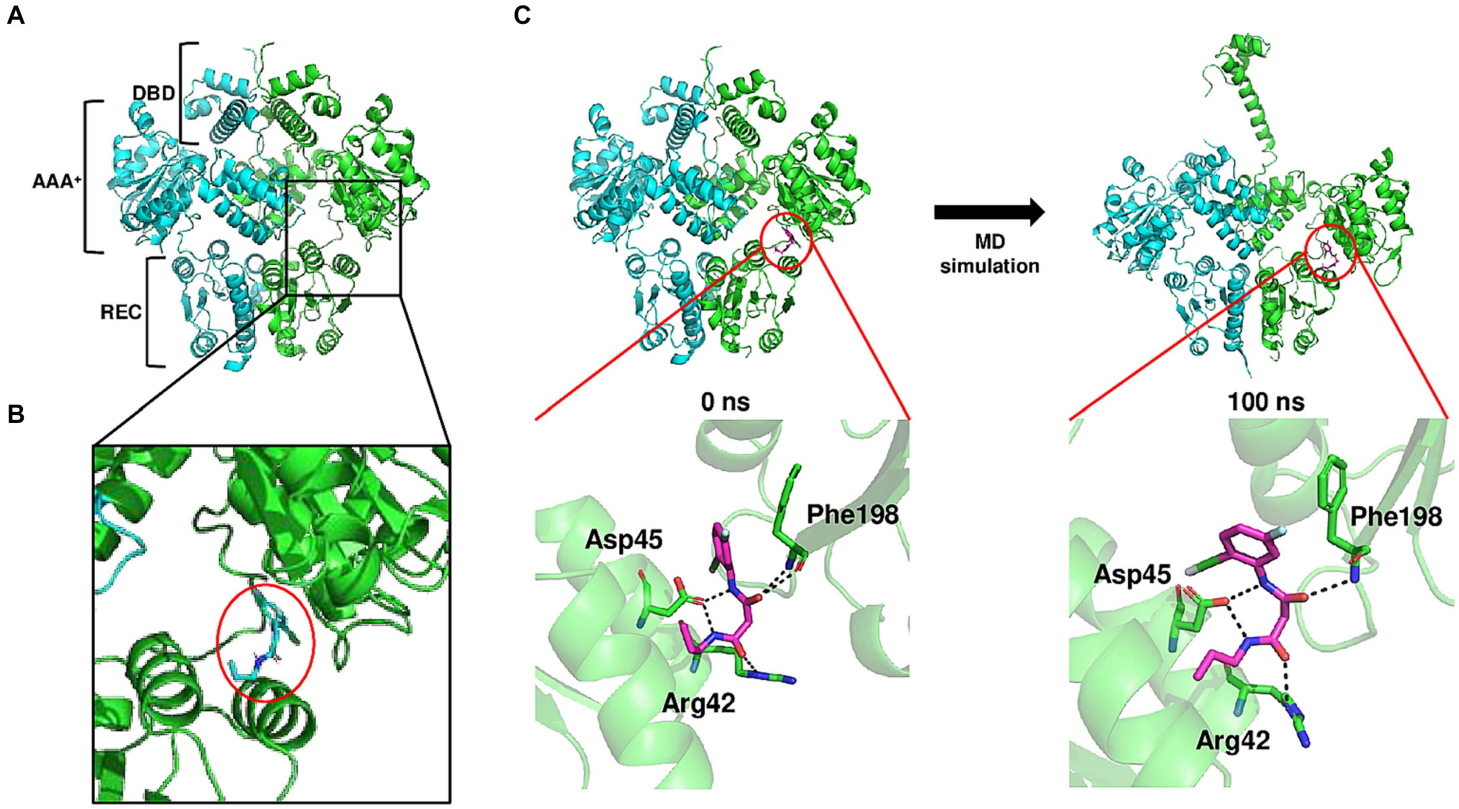
Conformational change of BrpR upon the interaction with BFstatin. **(A)** Molecular structure of BrpR dimer was predicted using the AlphaFold2 algorithm. DBD, DNA-binding domain; AAA^+^, ATPase associated with diverse cellular activities; REC, receiver domain. **(B)** Molecular docking of the BrpR dimer and BFstatin was performed using AutoDock Vina. The ligand-binding pocket of BrpR and docked BFstatin is presented in a close-up view. BFstatin is inside the red circle. **(C)** MD simulation with the docked BrpR-BFstatin complex was carried out using Gromacs software. The structure of the BrpR-BFstatin complex and close-up views of the ligand-binding pocket are presented vertically. Left, BrpR-BFstatin complex before MD simulation; Right, BrpR-BFstatin complex after 100 ns MD simulation.

Molecular modeling was used to identify how BFstatin interacts with BrpR and subsequently inhibits its DNA-binding activity. BrpR is expected to regulate transcription by dimerization as observed in its homolog VpsR of *Vibrio cholerae* (Chakrabortty et al., 2022). Thus, the molecular structure of the BrpR dimer was predicted using AlphaFold2, as depicted in Figure 4A. Similar to VpsR (Chakrabortty et al., 2022), BrpR consists of three domains: N-terminal receiver domain (REC), central AAA^+^ domain, and C-terminal helix-turn-helix DNA-binding domain (DBD). Molecular docking of the BrpR dimer and BFstatin was performed in a simulated environment using AutoDock Vina. As shown in Figure 4B, BFstatin was predicted to interact with the ligand-binding pocket of BrpR between the REC and AAA^+^ domains. To analyze the structural change of BrpR upon the interaction with BFstatin, the MD simulation was carried out with the docked BrpR-BFstatin complex. BrpR remained interacting with BFstatin without separation for 100 ns, indicating a strong interaction between BrpR and BFstatin (Figure 4C). Additionally, a structural change in BrpR, especially in the DBD, occurred by the interaction with BFstatin (Figure 4C). The structural change possibly contributed to the BFstatin inhibition of the DNA-binding activity of BrpR (Figures 3B,C).

### 3.5 BFstatin affects the expression of the BrpR regulon

**Figure 5:**
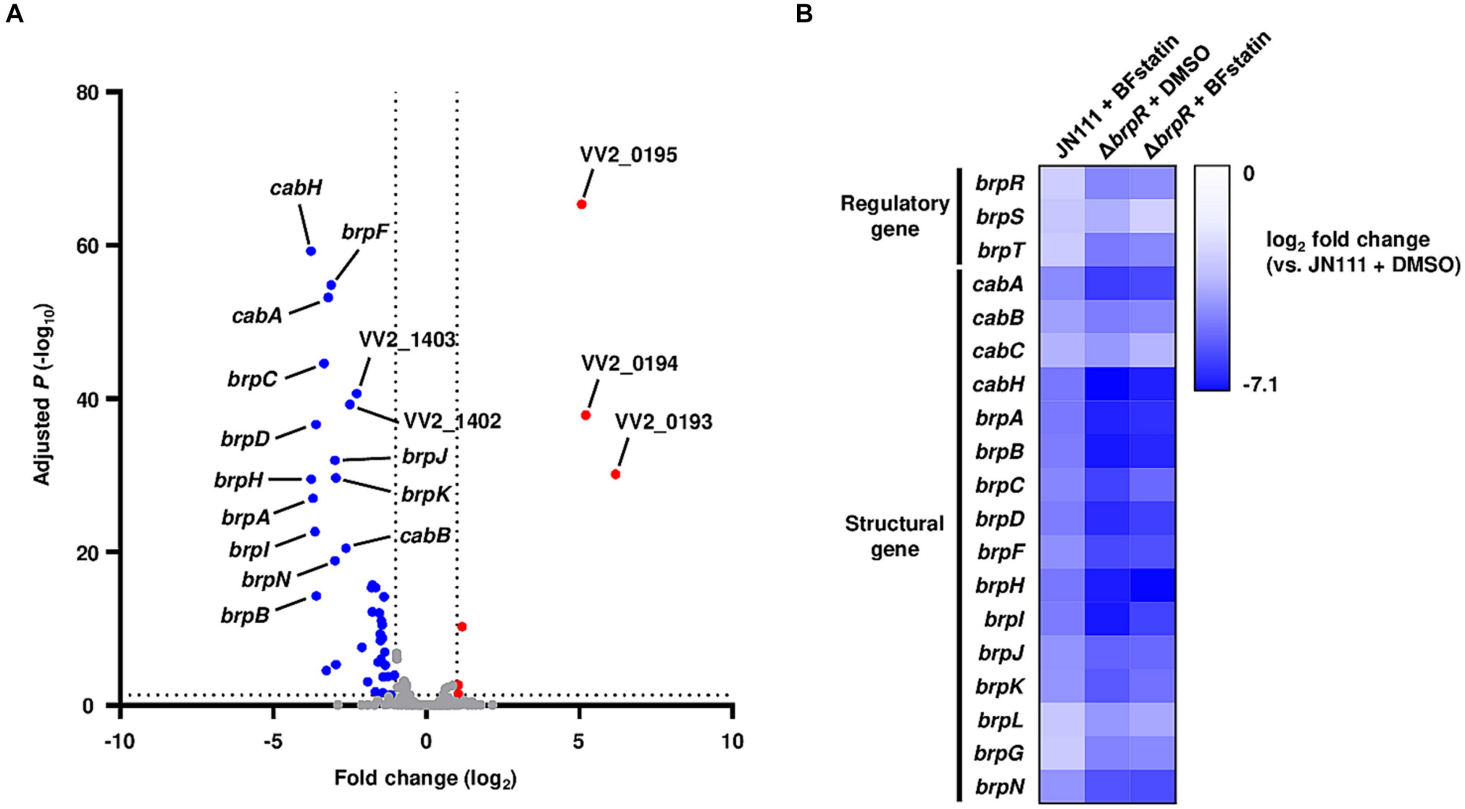
Effects of BFstatin on the gene expression of *V. vulnificus*. **(A)** The *V. vulnificus* JN111 genes differentially expressed by BFstatin are visualized in a volcano plot. The gray dashed lines represent cutoffs for differential expression of |fold change| >2 and adjusted *p*-value <0.05. The blue and red dots indicate the genes differentially down-regulated and up-regulated by the addition of 20  μM BFstatin, respectively. **(B)** Expression fold changes of the regulatory and structural genes of the BrpR regulon in *V. vulnificus* JN111 treated with BFstatin, Δ*brpR* treated with DMSO, and Δ*brpR* treated with BFstatin relative to those in JN111 treated with DMSO are displayed as a heatmap. The comparative analyses of the gene expression were performed with biological triplicates.

Because BFstatin inhibited BrpR from binding to its target promoter DNA, BFstatin might affect the expression of the BrpR regulon. To examine whether BFstatin affects the expression of the BrpR regulon, transcriptomic analyses were performed. By comparing transcriptomes of the BFstatin-treated and DMSO-treated (control) *V. vulnificus*, the genes expressed differentially upon the addition of 20 μM BFstatin were identified. BFstatin up-regulated a total of 6 genes including VV2_0193-0195 operon encoding transporter proteins (Figure 5A; Supplementary material S3). On the other hand, BFstatin down-regulated 40 genes comprising BrpR regulon involved in biofilm formation, such as the *cabABC* operon and *brp* locus (Figure 5A; Supplementary material S3).

Figure 5B shows the fold changes of the BrpR-regulated biofilm genes in the BFstatin-treated *V. vulnificus*, DMSO-treated Δ*brpR*, and BFstatin-treated Δ*brpR* relative to DMSO-treated *V. vulnificus* (see Supplementary materials S3–S5 for details). As expected from the observation that BrpR activates its own transcription by directly binding to the promoter DNA of *brpR* (Supplementary Figure S1B), the expression level of *brpR* was also down-regulated by BFstatin (Figure 5B). The result indicated that the effects of BFstatin on the transcription of biofilm genes can be further amplified by reducing the BrpR expression. In addition, the expression levels of the biofilm genes in DMSO-treated Δ*brpR* and BFstatin-treated Δ*brpR* were comparable (Figure 5B). The result suggested that BFstatin has no extra effects on the expression of the biofilm genes other than the inhibition of the BrpR activity.

### 3.6 BFstatin interrupts the development of the biofilm matrix

**Figure 6:**
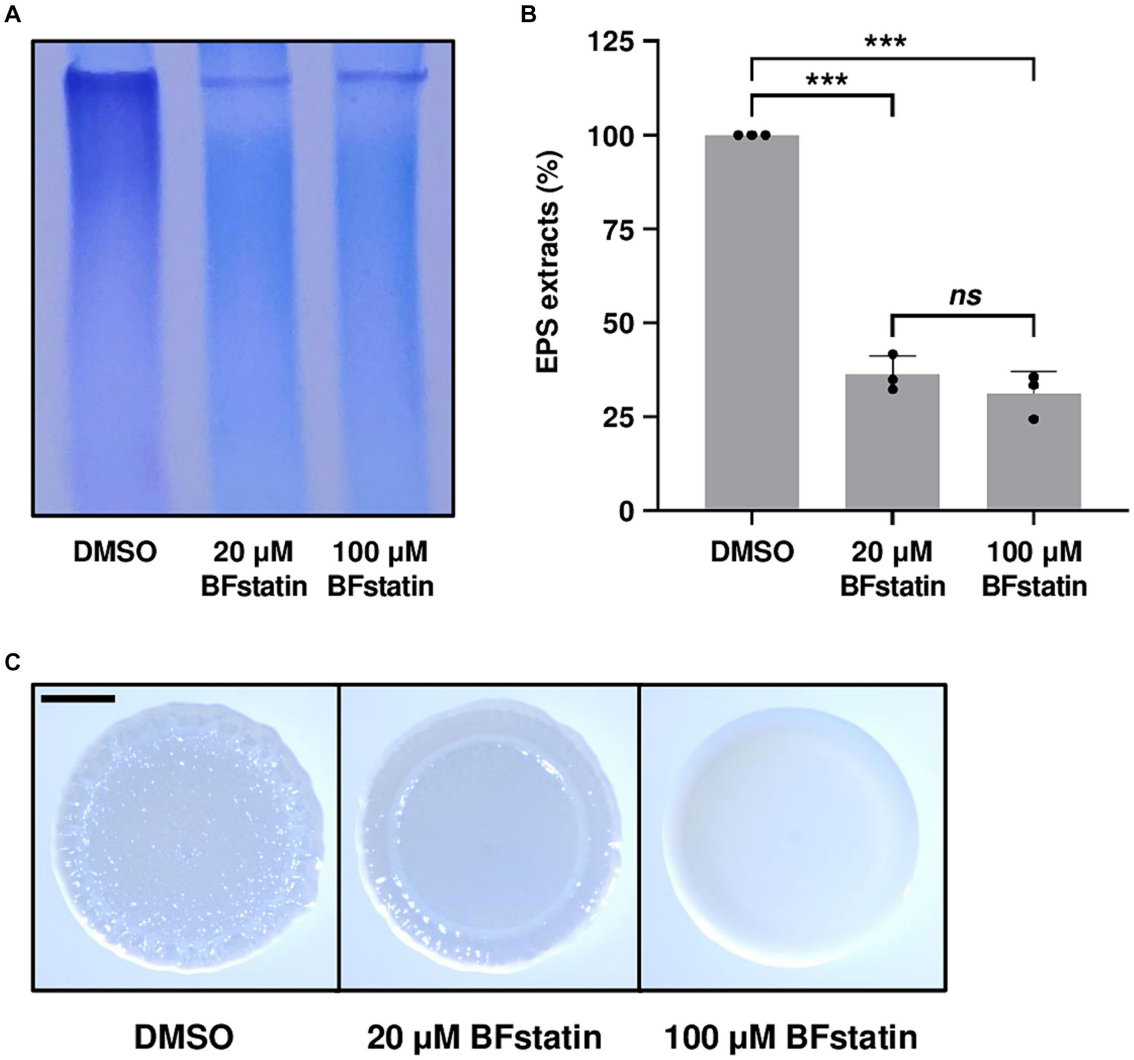
Effects of BFstatin on the biofilm matrix development of *V. vulnificus*. **(A)** EPS extracts were prepared from *V. vulnificus* JN111 grown on LBS agar supplemented with 0.02% arabinose and either 20 or 100 μM BFstatin or 0.1% DMSO (control), and resolved on a 4% polyacrylamide gel by SDS-PAGE. The gel containing EPS was stained with stains-all. Then the gel representing the mean result from at least three independent experiments was photographed using a mobile camera. **(B)** Relative EPS extracts (%) were quantified from the intensity of each lane of the gel, and the extract of the control group was set as 100% in each experiment. Error bars represent the SD from biological triplicates. Statistical significance was determined by the student’s *t*-test (^***^p < 0.0005; ns, not significant). **(C)** *V. vulnificus* JN111 was spotted onto VFMG agar supplemented with 0.02% arabinose and either 20 or 100 μM BFstatin or 0.1% DMSO (control), then incubated for 24 h. Each colony representing the mean rugosity from at least three independent experiments was visualized using a stereomicroscope. All images are shown at the same scale, and a 1 mm scale bar is shown on the image of the control.

Since BrpR regulates the genes involved in the biosynthesis of biofilm matrix components such as EPS, the effect of BFstatin on the EPS production of *V. vulnificus* was investigated using SDS-PAGE. When treated with BFstatin, the amounts of EPS extracted from *V. vulnificus* considerably diminished (Figures 6A,B), demonstrating that BFstatin inhibited the production of EPS. Because changes in the biofilm matrix components alter the colony shape (Yildiz and Visick, 2009; Serra et al., 2013), the effect of BFstatin on the colony morphology was also further examined. In the absence of BFstatin, the colony morphology of *V. vulnificus* was rugose, confirming the presence of biofilm matrix components (Figure 6C). However, when BFstatin was treated, the rugosity of the *V. vulnificus* colony was decreased to smoothness and the level of the decreased rugosity was proportional to the concentration of BFstatin treated. These results indicated that BFstatin interrupts the development of biofilm matrix in a dose-dependent manner by hindering the production of the matrix components such as EPS.

### 3.7 BFstatin suppresses the biofilm formation of *Vibrio vulnificus*

**Figure 7:**
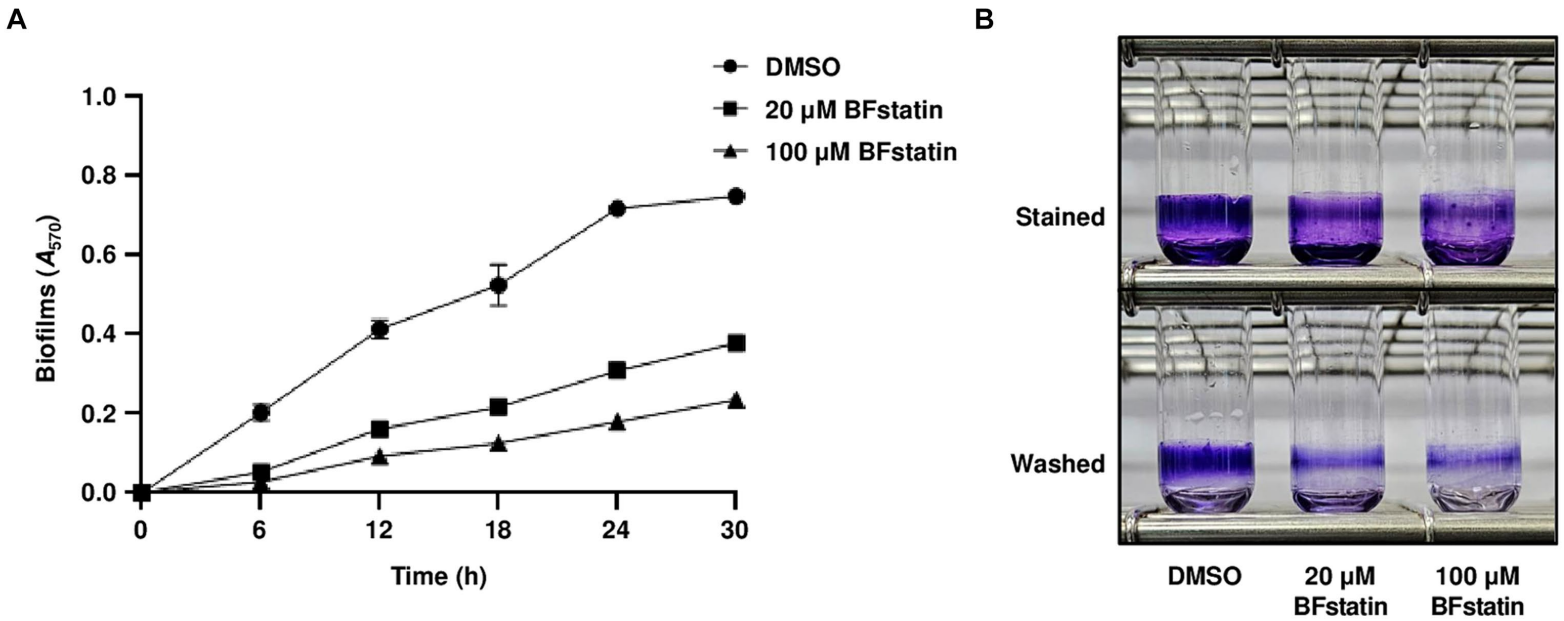
Effects of BFstatin on the biofilm formation of *V. vulnificus*. **(A)** Biofilms of *V. vulnificus* JN111 were grown in VFMG supplemented with 0.01% arabinose and either 20 or 100 μM BFstatin or 2% DMSO (control) in 96-well polystyrene microtiter plates for 6, 12, 18, 24, and 30 h, then stained with 1% crystal violet. The crystal violets were eluted and their *A*_570_ were determined to quantify the biofilms. Error bars represent the SD from biological triplicates. **(B)** To visualize the biofilm, biofilms were grown in glass test tubes for 24 h and stained with 1% crystal violet. The stained biofilms were then washed with vibration in PBS to remove loosely attached cells. Each test tube representing the mean result from at least three independent experiments was photographed using a mobile camera. Stained, photographed after being stained with crystal violet; Washed, photographed after being washed with PBS.

Considering that BFstatin interrupted the development of biofilm matrix, the effect of BFstatin on biofilm formation was investigated. After the addition of different concentrations of BFstatin to *V. vulnificus*, their biofilm levels were observed for up to 30 h using 96-well polystyrene microtiter plates. At every time point, the biofilm formation of *V. vulnificus* with 20 μM BFstatin significantly decreased when compared with that of the bacteria without BFstatin (Figure 7A). Moreover, the biofilm level of *V. vulnificus* with 100 μM BFstatin diminished when further compared with that of the bacteria with 20 μM BFstatin (Figure 7A). The effect of BFstatin inhibiting biofilm formation lasted for at least 30 h (Figure 7A), showing its long-term efficacy for inhibition. Biofilms grown for 24 h were further visualized in large scales using glass test tubes. As shown in Figure 7B, defects in biofilm development upon BFstatin treatment were visually confirmed. These results demonstrated that BFstatin suppresses the biofilm formation of *V. vulnificus* in a dose-dependent manner, and the suppressive effect is not temporary.

## 4 Discussion

For biofilm development, *V. vulnificus* expresses diverse genes that are regulated by the master regulator BrpR. Therefore, inhibiting the activity of BrpR can diminish the expression of the genes, thereby reducing biofilm formation. In this study, high-throughput screening of 6,750 compounds with various molecular structures was performed to identify small molecules that significantly reduce the activity of BrpR (Figure 1). A small molecule, whose molecular weight is 272.71 g/mol, was effective in inhibiting the activity of BrpR even at low concentrations in the micromolar range (Figures 2A,B). The small molecule was named BFstatin, with the meaning of repressing biofilm formation. Most chemicals currently used to treat bacterial biofilms interfere with the growth of bacteria, so the emergence and dominance of resistant strains are inevitable (Smith and Coast, 2002; Clatworthy et al., 2007; Koo et al., 2017). However, BFstatin did not impede the growth of *V. vulnificus* (Figure 2C), generating less selective pressure for the dominance of resistance. Additionally, BFstatin did not show cytotoxicity to the human epithelial cell line (Figure 2D), expanding its possible application in the seafood industry.

As a regulator, BrpR binds to the promoter DNAs and activates the transcription of downstream biofilm genes of *V. vulnificus* (Hwang et al., 2021). BFstatin interacted directly with BrpR and decreased its binding to the target promoter DNAs (Figure 3), thereby inhibiting the expression of the downstream biofilm genes. BFstatin could be effective in preventing biofilm formation because it hinders the expression of the biofilm genes rather than the activity of the expressed gene products. The interaction between BFstatin and BrpR was further investigated by molecular docking and MD simulation. Molecular docking showed that BFstatin could interact with the ligand-binding pocket between the REC and AAA^+^ domains of BrpR (Figures 4A,B). MD simulation further illustrated that the interaction with BFstatin induced the structural change of BrpR, particularly in the DBD (Figure 4C), proposing that BFstatin inhibition of the BrpR binding to the target promoter DNAs was mediated through the structural change of the DBD.

Transcriptomic analyses showed that BFstatin reduced the expression of BrpR regulon, especially the biofilm genes such as the *cabABC* operon and *brp* locus (Figure 5). By reducing the expression of the BrpR regulon, BFstatin diminished the production of biofilm matrix components which are required for the successful development of biofilms (Figure 6). In addition, BFstatin showed a consistent suppressive effect on biofilm formation for up to 30 h (Figure 7), revealing its long-term efficacy for biofilm inhibition. Since biofilms generally inhibit the access of antibiotics to target bacteria (Ciofu et al., 2017; Sharma et al., 2019), BFstatin could allow better effectiveness of antibiotics against *V. vulnificus* by suppressing biofilm formation. Surprisingly, BFstatin significantly changed the expression of the VV2_0193-0195, VV2_1402, and VV2_1403 genes which are not involved in BrpR regulon (Figure 5A) (Hwang et al., 2021). The expression of VV2_0193-0195, encoding subunits of efflux resistance-nodulation-division (RND) transporter that pumps out molecules inducing stress, was increased by BFstatin (Figure 5A; Supplementary material S3) (Nikaido and Takatsuka, 2009). *V. vulnificus* may have recognized BFstatin as a stress-inducing molecule and increased expression of the efflux RND transporter, which could further play a role in the resistance of *V. vulnificus* to other molecules inducing stress (Alvarez-Ortega et al., 2013). The expression of VV2_1402 and VV2_1403, encoding a drug/metabolite family transporter that possibly translocates substrates for biofilm development and a diguanylate cyclase that synthesizes c-di-GMP, respectively, was decreased by BFstatin (Figure 5A; Supplementary material S3) (Jack et al., 2001; Whiteley and Lee, 2015). The decreased expression of VV2_1402 and VV2_1403 in *V. vulnificus* may have been accompanied with the suppressed biofilm formation by BFstatin.

Other than antibiotic treatment, numerous methods are currently being applied to control microbial biofilms (Srinivasan et al., 2021). One way is to remove the biofilms by physical-mechanical methods, such as using high-velocity spray or jet irrigators (Kato et al., 2012; Fabbri et al., 2016). Also, biochemical methods such as EPS-degrading enzymes are applied to decompose the matrix and thus to remove the biofilms (Gunn et al., 2016). Although these methods are effective in temporarily removing pre-formed biofilms, the possibility that biofilms will form again is still high (Subramani and Hoek, 2010; Ohsumi et al., 2015). Unlike these removals of pre-formed biofilms, BFstatin prevents biofilm formation by inhibiting the expression of genes required for matrix development. Therefore, the addition of BFstatin to the biofilm-removing methods could prevent the re-formation of biofilms. The combined methods can be used in seafood industries, such as oyster farms, to remove the *V. vulnificus* biofilms effectively.

**Figure 8:**
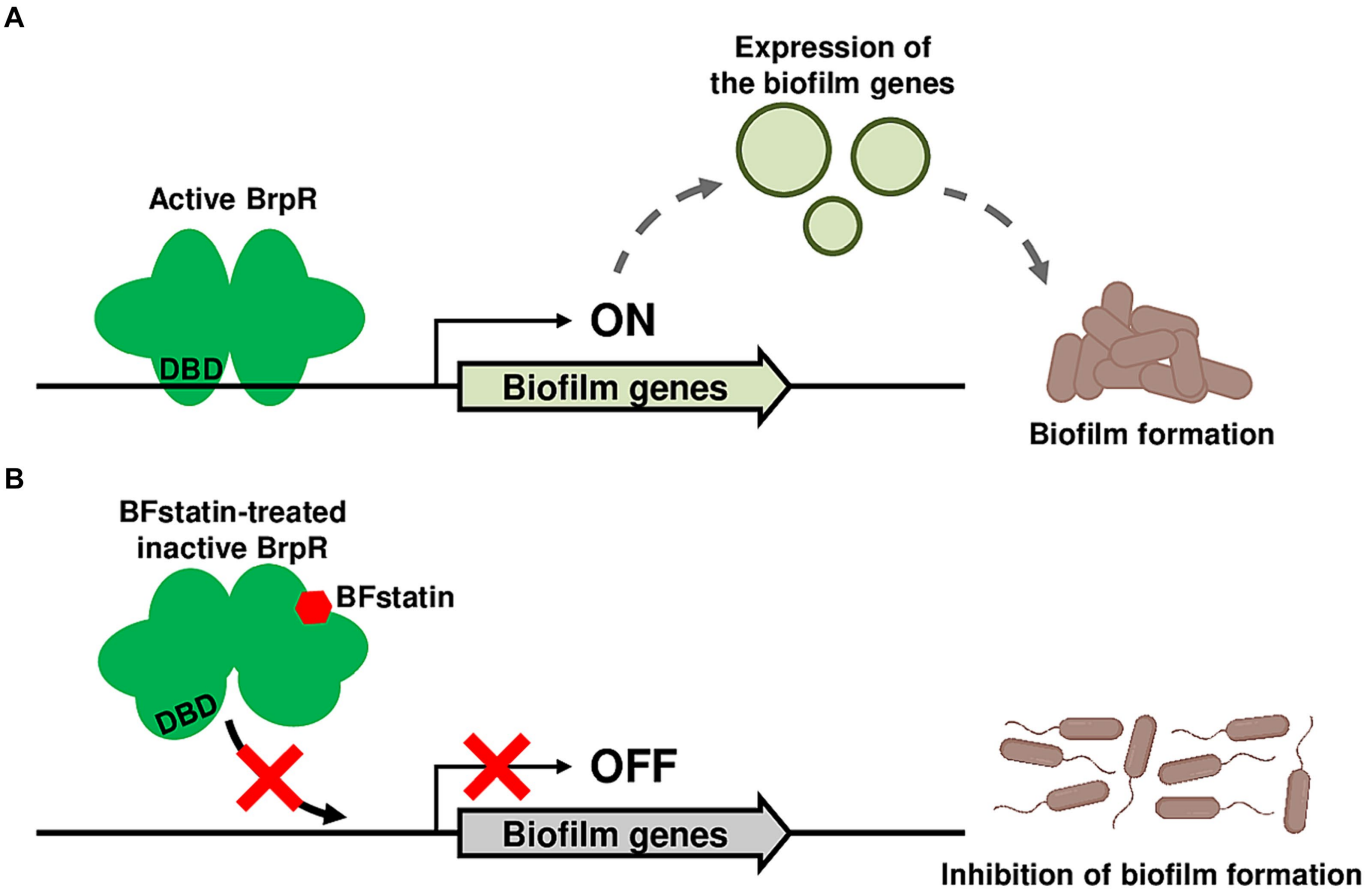
Proposed mechanism for the BFstatin-mediated inhibition of the *V. vulnificus* biofilm formation. **(A)** Active BrpR binds to its target promoter DNA using the DNA-binding domain, and the expression of biofilm genes is induced, leading to the formation of biofilms. **(B)** In contrast, BFstatin modifies the molecular structure of BrpR and thereby inhibits its binding to the target promoter DNA. Consequently, the expression of biofilm genes is not induced, resulting in the inhibition of biofilm formation. DBD, DNA-binding domain.

Many bacterial pathogens including *E. coli*, *Pseudomonas aeruginosa*, and *V. cholerae* form biofilms, which are closely linked to their pathogenicity (Parsek and Singh, 2003; Joo and Otto, 2012; Sharma et al., 2016; Silva and Benitez, 2016; Wang et al., 2023). Therefore, identifying small molecules inhibiting biofilm formation of the pathogens could be useful in controlling their infections. In this study, a small molecule BFstatin that selectively inhibits the activity of the transcriptional regulator BrpR of a devastating pathogen *V. vulnificus* was identified as an anti-biofilm agent. As depicted in Figure 8, BFstatin directly interacts with BrpR and modifies its molecular structure, especially in the DBD. Thereby, BFstatin hinders the binding of BrpR to the target promoter DNAs, leading to reduced expression of biofilm matrix genes. Consequently, BFstatin inhibited the biofilm formation of *V. vulnificus* by diminishing the production of biofilm matrix components. Since BFstatin did not show any bacteriostatic activities, it could be used to control *V. vulnificus* biofilms without inducing the dominance of resistant strains.

## Data Availability

The datasets presented in this study can be found in online repositories. The names of the repository/repositories and accession number(s) can be found in the article/Supplementary material.

## Footnotes

^1^www.chembank.org

^2^https://www.ncbi.nlm.nih.gov/bioproject
