# Supplementary material for: Identification and characterization of a small molecule BFstatin inhibiting BrpR, the transcriptional regulator for biofilm formation of Vibrio vulnificus
Source: Front Microbiol. 2024 Sep 9;15:1468567. doi: 10.3389/fmicb.2024.1468567 (PMC11416940; doi:10.3389/fmicb.2024.1468567)
Supplement: Supplementary file 6 [file Image_1.PDF]

Supplementary Material

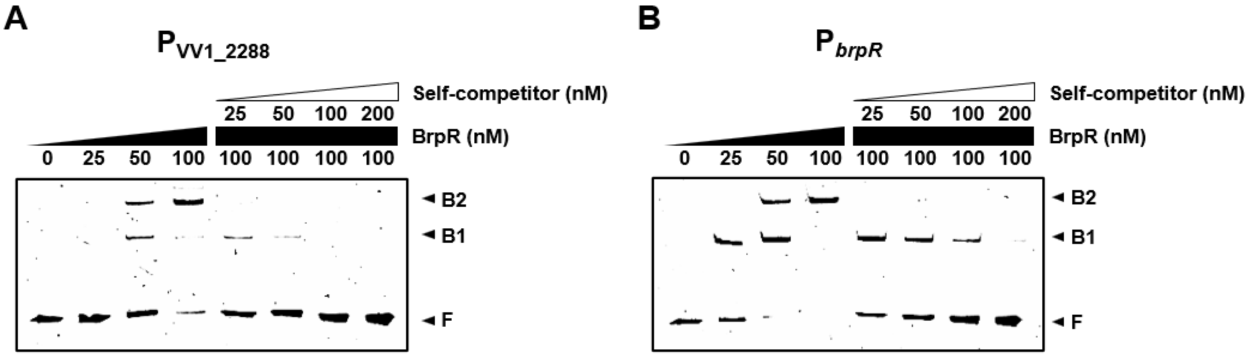

Supplementary Figure S1. Direct binding of BrpR to the promoter DNAs of VV1_2288 and *brpR*. (A, B) The 6-FAM labeled VV1_2288 (**A**) and *brpR* (**B**) promoter DNA probes (5 nM) were incubated with increasing amounts of BrpR (from 0 to 100 nM) as indicated. For competition analysis, the same but unlabeled DNA probes were used as self-competitors. Increasing amounts of self-competitors (from 0 to 200 nM) were added as indicated to the reaction mixtures before the addition of 100 nM BrpR. Each gel representing the mean result from at least three independent experiments was photographed using the ChemiDoc Touch Imaging System. B1&B2, BrpR-bound DNA; F, free DNA.
