# Supplementary material for: Identification and characterization of a small molecule BFstatin inhibiting BrpR, the transcriptional regulator for biofilm formation of Vibrio vulnificus
Source: Front Microbiol. 2024 Sep 9;15:1468567. doi: 10.3389/fmicb.2024.1468567 (PMC11416940; doi:10.3389/fmicb.2024.1468567)
Supplement: Supplementary file 7 [file Table_1.DOCX]

Supplementary Material

**Supplementary Table S1. Bacterial strains and plasmids used in this study.**

| Strain or plasmid | Relevant characteristics*^a^* | Reference of source |
| --- | --- | --- |
| Bacterial strains | | |
| *V. vulnificus* | | |
| CMCP6 | Wild type; clinical isolate; virulent | Laboratory collection |
| JN131 | CMCP6 with Δ*brpR* | (Hwang et al., 2021) |
| JN111 | CMCP6 with P_BAD_-*dcpA*, model strain | (Park et al., 2015) |
| JN131D | JN111 with Δ*brpR* | (Hwang et al., 2020) |
| *E. coli* | | |
| DH5α | *supE44 ΔlacU169* (*Φ80 lacZ Δ*M15) *hsdR17 recA1 endA1 gyrA96 thi-1 relAI* | Laboratory collection |
| BL21 (DE3) | *F*^-^, *ompT*, *hsdS* (r_B_^-^, m_B_^-^), *gal dcm* (DE3) | Laboratory collection |
| Plasmids | | |
| pJK1113 | pKS1101 with *nptI*; Ap^r^ Km^r^ | (Lim et al., 2014) |
| pJN1601 | pJK1113 with *brpR*; Ap^r^ Km^r^ | (Hwang et al., 2020) |
| pBBR-lux | Broad host range vector with promoterless *luxCDABE*; Cm^r^ | (Lenz et al., 2004) |
| pSH2103 | pBBR-lux with P_VV1_2288_; Cm^r^ | This study |
| pJN1606 | pBBR-lux with P*_btpT_*; Cm^r^ | This study |
| pSH1820 | pET-28a(+) with *brpR*; Km^r^ | (Hwang et al., 2021) |

*^a^*Ap^r^, ampicillin-resistant; Km^r^, kanamycin-resistant; Cm^r^, chloramphenicol-resistant.

**References**

Hwang S H, Im H, Choi S H (2021). A Master Regulator BrpR Coordinates the Expression of Multiple Loci for Robust Biofilm and Rugose Colony Development in *Vibrio vulnificus*. Frontiers in Microbiology, 12

Hwang S H, Park J H, Lee B, Choi S H (2020). A Regulatory Network Controls *cabABC* Expression Leading to Biofilm and Rugose Colony Development in *Vibrio vulnificus*. Frontiers in Microbiology, 10

Lenz D H, Mok K C, Lilley B N, Kulkarni R V, Wingreen N S, Bassler B L (2004). The small RNA chaperone Hfq and multiple small RNAs control quorum sensing in *Vibrio harveyi* and *Vibrio cholerae*. Cell, 118(1): 69-82

Lim J G, Bang Y J, Choi S H (2014). Characterization of the *Vibrio vulnificus* 1-Cys Peroxiredoxin Prx3 and Regulation of Its Expression by the Fe-S Cluster Regulator IscR in Response to Oxidative Stress and Iron Starvation. Journal of Biological Chemistry, 289(52): 36263-36274

Park J H, Jo Y, Jang S Y, Kwon H, Iriecurrency Y, Parsek M R, Kim M H, Choi S H (2015). The *cabABC* Operon Essential for Biofilm and Rugose Colony Development in *Vibrio vulnificus*. Plos Pathogens, 11(9)
