# Supplementary material for: Identification and characterization of a small molecule BFstatin inhibiting BrpR, the transcriptional regulator for biofilm formation of Vibrio vulnificus
Source: Front Microbiol. 2024 Sep 9;15:1468567. doi: 10.3389/fmicb.2024.1468567 (PMC11416940; doi:10.3389/fmicb.2024.1468567)
Supplement: Supplementary file 8 [file Table_2.DOCX]

Supplementary Material

**Supplementary Table S2. Oligonucleotides used in this study.**

| Oligonucleotide | Oligonucleotide sequence (5’ →3’)*^a^* | Use |
| --- | --- | --- |
| For luminescence assay | | |
| P12288_lux_F | ATGAGCTCCGAGATCCGACCACGTAAC | Amplification of VV1_2288 upstream region to construct pSH2103 |
| P12288_lux_R | TAGGATCCCCACCCAAATCAGAGCCTG |  |
| PBRPT_lux_F | ATGAGCTCGCTCTAATTTGGGCTAAGGAACA | Amplification of *brpT* upstream region to construct pJN1606 |
| PBRPT_lux_R | TAGGATCCCATCCCAGTTTCAGCCATCAG |  |
| For EMSA | | |
| BRPL_UP_F | GTCTTACCAAGCTAGACCCGT | Amplification of *brpL* upstream region |
| BRPL_UP_R | TGATGATATTGACTGCAAGCGT |  |
| 12288_UP_F | GCATCAGTCTAAACACCGCAC | Amplification of VV1_2288 upstream region |
| 12288_UP_R | GCATCAGTCTAAACACCGCAC |  |
| BRPR_UP_F | GCATCAGTCTAAACACCGCAC | Amplification of *brpR* upstream region |
| BRPR_UP_R | TGGCGATGGCTCTAAAGTTGT |  |

*^a^*Regions of oligonucleotides not complementary to the corresponding genes are underlined.
